# Monolayer-graphene-based broadband and wide-angle perfect absorption structures in the near infrared

**DOI:** 10.1038/s41598-018-32052-7

**Published:** 2018-09-12

**Authors:** Yansong Fan, Chucai Guo, Zhihong Zhu, Wei Xu, Fan Wu, Xiaodong Yuan, Shiqiao Qin

**Affiliations:** 1 College of Advanced Interdisciplinary Studies, National University of Defense Technology, Changsha, 410073 China; 2 State Key Laboratory of High Performance Computing, National University of Defense Technology, Changsha, 410073 China

## Abstract

Broadband optical absorption structures in the near infrared by coupling monolayer-graphene with periodical metal structures are proposed and demonstrated numerically. Optical absorption of graphene with over-50%-absorption bandwidth up to hundreds of nanometer caused by magnetic dipole resonances and magnetic coupling effect are investigated in detail, and the demonstrated bandwidths are one order higher than those caused by dielectric guiding mode resonances. In addition, the influences of geometrical parameters of structures are fully analyzed and these demonstrated structures show angular-insensitive absorption for oblique incidence in a large angular range. The demonstrated absorption structures in this work provide new design ideas in the realization of advanced graphene-based optoelectronic devices.

## Introduction

Graphene has been widely applied in different kinds of optoelectronic devices due to its excellent physical properties^1–3^, such as stable optical response in broad spectrum^4^ and ultra-high carrier mobility^5^. However, the absorption of monolayer graphene towards normally incident light is too low^6^, which limits its application in optoelectronic devices. Therefore, many methods have been put forward to enhance the absorption of graphene. In the mid-infrared to THz range, graphene absorption is normally enhanced by exciting surface plasmonic resonance of graphene^7–13^. In the visible to near-infrared range, graphene does not support surface plasmonic response, and many resonant structures have been utilized to couple with graphene to enhance the graphene absorption^14–24^ or realize perfect absorption of input light^25–35^.

Recently, we experimentally demonstrated peak absorption over 99% for monolayer graphene coupled with one-dimensional^30^ and two-dimensional^31^ dielectric periodical structures. However, the absorption bandwidths of these demonstrated structures are less than 20 nm, and the absorption spectra of these structures are sensitive to the incident angle of the input light. As for some graphene-based optoelectronic devices, broadband graphene absorption with incident angle independence are highly desirable.

In this paper, we propose to take advantage of metal-insulation-metal (MIM) resonators which have been reported to achieve broadband absorption^36–39^, to design graphene-based broadband and wide-angle perfect absorption structures in the near infrared. Research results show that the bandwidth of graphene absorption over 50% can exceed 200 nm at center wavelength near 1550 nm by using magnetic dipole resonances^40–42^ or magnetic coupling effects^43^. Meanwhile, all these proposed structures could work in a large incident angle range. The demonstrated structures would be of valuable applications in advanced graphene-based optoelectronic devices.

## Results and Discussion

**Figure 1:**
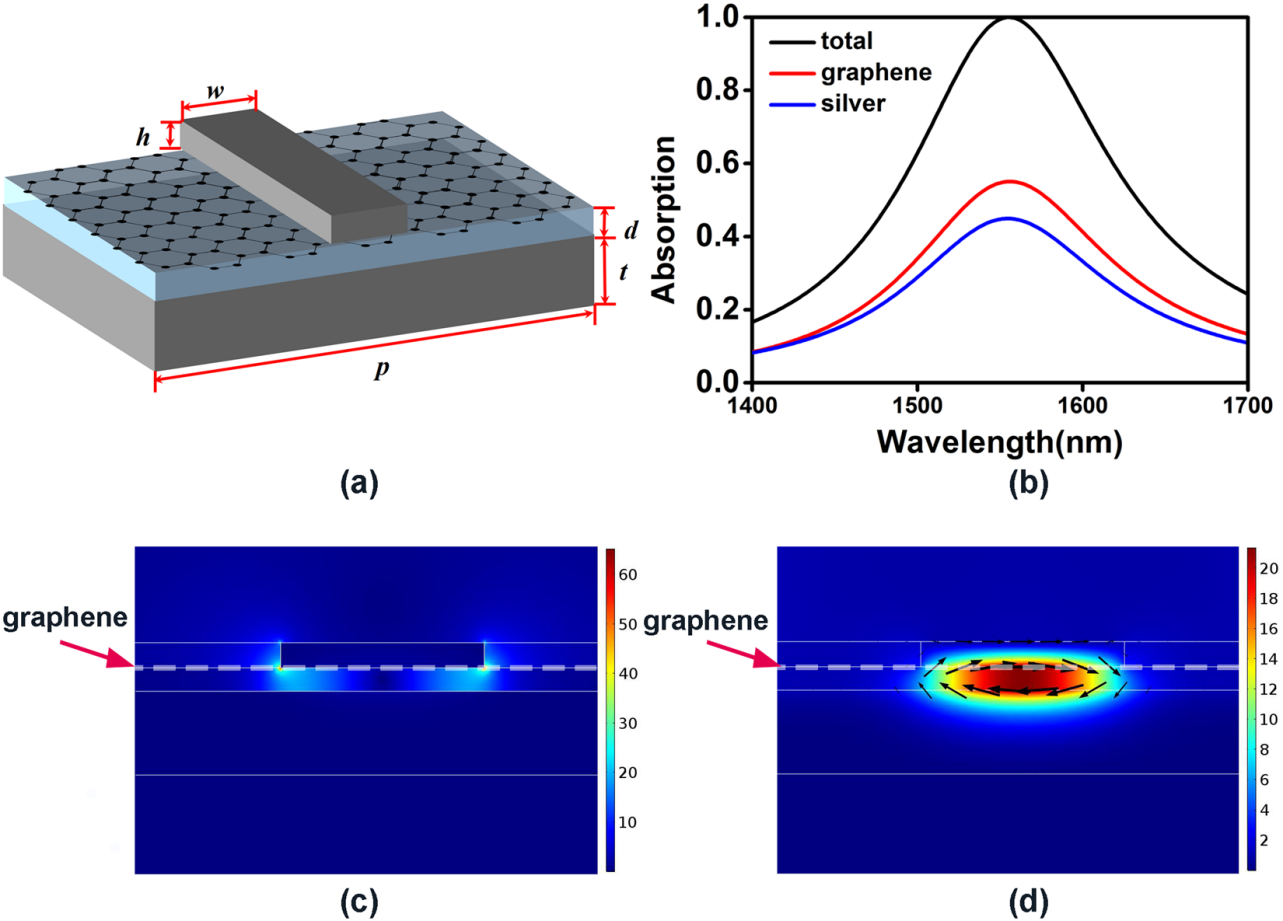
(**a**) Schematic of the unit cell of the graphene-based perfect absorption structure using magnetic dipole resonance. (**b**) Simulated total absorption spectrum and absorption spectra of the monolayer graphene and the metal. The electric and magnetic field distribution of the 1D metal grating structure at the resonant wavelength: (**c**) Normalized electric field at 1556 nm. (**d**) Normalized magnetic field at 1556 nm, where the black arrows represent electric displacement. The white dashed line marks the position of graphene in (**c**) and (**d**).

The schematic image of the unit cell of our proposed perfect absorption structure using magnetic dipole resonance is shown in Fig. 1(a). A monolayer graphene is sandwiched between a silver grating and a lossless alumina layer on top of a silver reflection mirror. The thickness of grating (*h*), dielectric layer (*d*) and reflection layer (*t*) are set as 30 nm, 28 nm and 100 nm, respectively. The width (*w*) and the period (p) of grating is 243 nm and 1000 nm.

Figure 1(b) shows the absorption spectra of the designed structure for traverse-magnetic (TM) polarization. As shown in Fig. 1(b), the absorption peak of the structure is located at 1556 nm with total absorption over 99.9%, where the absorption of graphene and silver (including silver grating and silver mirror) are 55.1% and 44.9%, respectively. The full width at half maximum (FWHM) of total absorption is beyond 150 nm, one order higher than that of graphene-dielectric coupling structures^15–18^, and the over-50% absorption bandwidth of graphene is about 46 nm.

To analyze the physical mechanisms of the resonant mode related to the absorption peak in Fig. 1(b), we investigate the electromagnetic field and electric displacement distributions of the resonant mode in Fig. 1(c,d). The electromagnetic fields have been normalized with incident wave, and the direction and size of black arrows represent the direction and magnitude of electric displacement. It can be found that around 1556 nm, the electric field is mostly concentrated around bottom corners of the grating and the magnetic field is highly confined in the dielectric layer between the silver grating and silver reflection mirror. The electric displacement are focused around grating bottom and metal mirror top with opposite direction. These features are corresponded to characteristics of magnetic dipole resonance. Moreover, the electric and magnetic fields around graphene are enhanced over 60 and 20 times, respectively. Such strong localized electrical and magnetic fields efficiently enhance the absorption of graphene.

**Figure 2:**
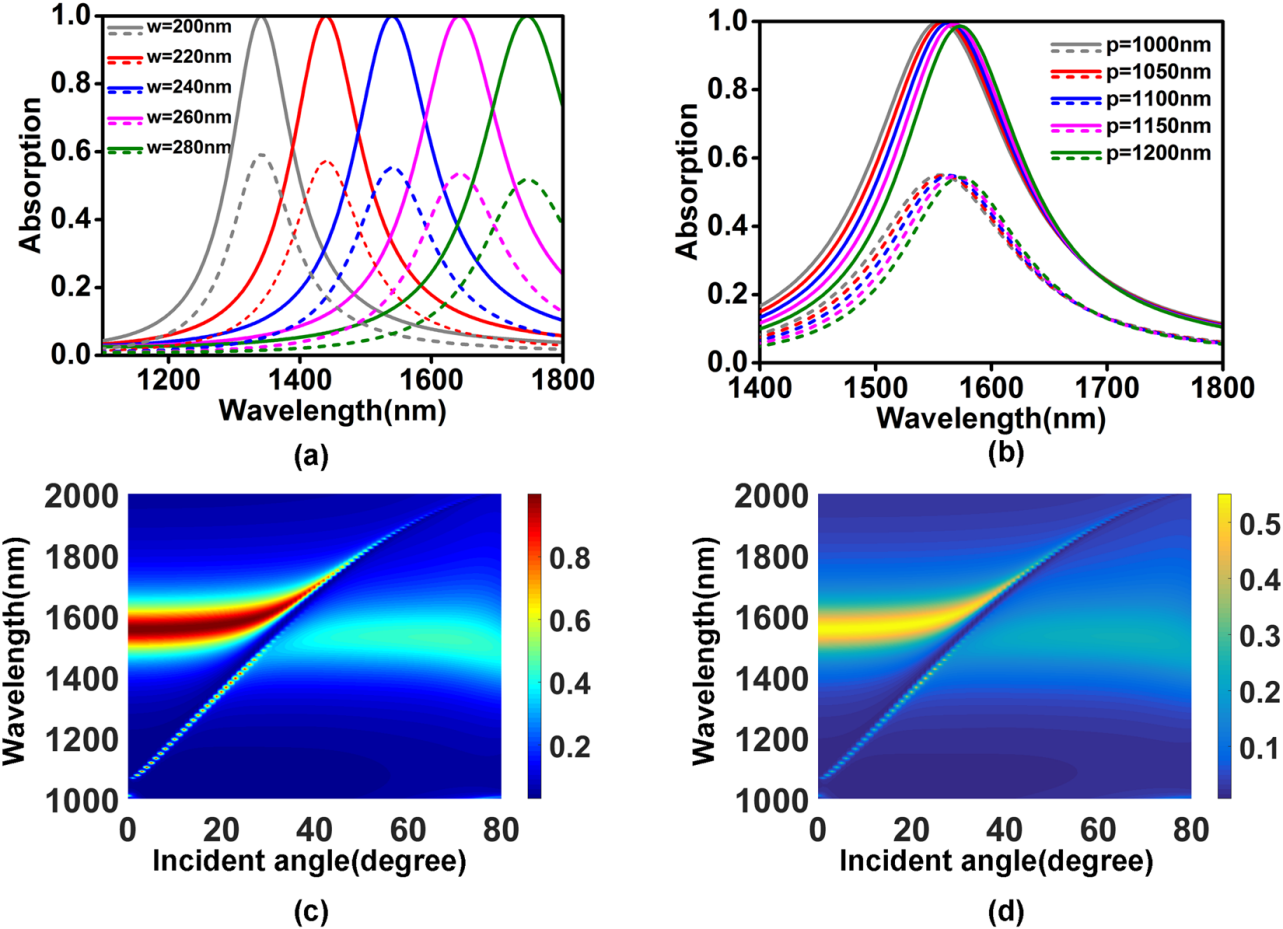
The absorption spectra of total structure and graphene as functions of (**a**) grating width (**b**) structure period. Absorption of (**c**) total structure and (**d**) graphene as functions of the wavelength and incident angle for TM polarization. Other geometric parameters are fixed as in Fig. 1.

Figure 2(a,b) show the influence of geometric parameters of the structure on the absorption spectra of total structure (solid lines) and graphene (dashed lines). As shown in Fig. 2(a,b), absorption peak wavelength of the structure increases along with the increase of the grating width, but it is insensitive to the grating period since the magnetic dipole resonance is a kind of local surface plasmon polarizations. Simulation results show that the peak absorption of the total structure is nearly unchanged and the peak absorption of the graphene decreases slightly along with the increase of the grating width. And the effect of the grating period on the peak absorption of the structure can be nearly ignored in the grating period range from 1000 nm to 1200 nm. The angle-dependent feature of the structure toward TM polarization is presented in Fig. 2(c,d). The absorption spectra from 1400 nm to 1800 nm are stable with the incident angle less than 20 degree. A propagating surface plasmon mode interacts with the magnetic dipole resonance when the light illuminates at the angle from 20 degree to 40 degree, leading to an asymmetric line-shape in the absorption spectrum.

**Figure 3:**
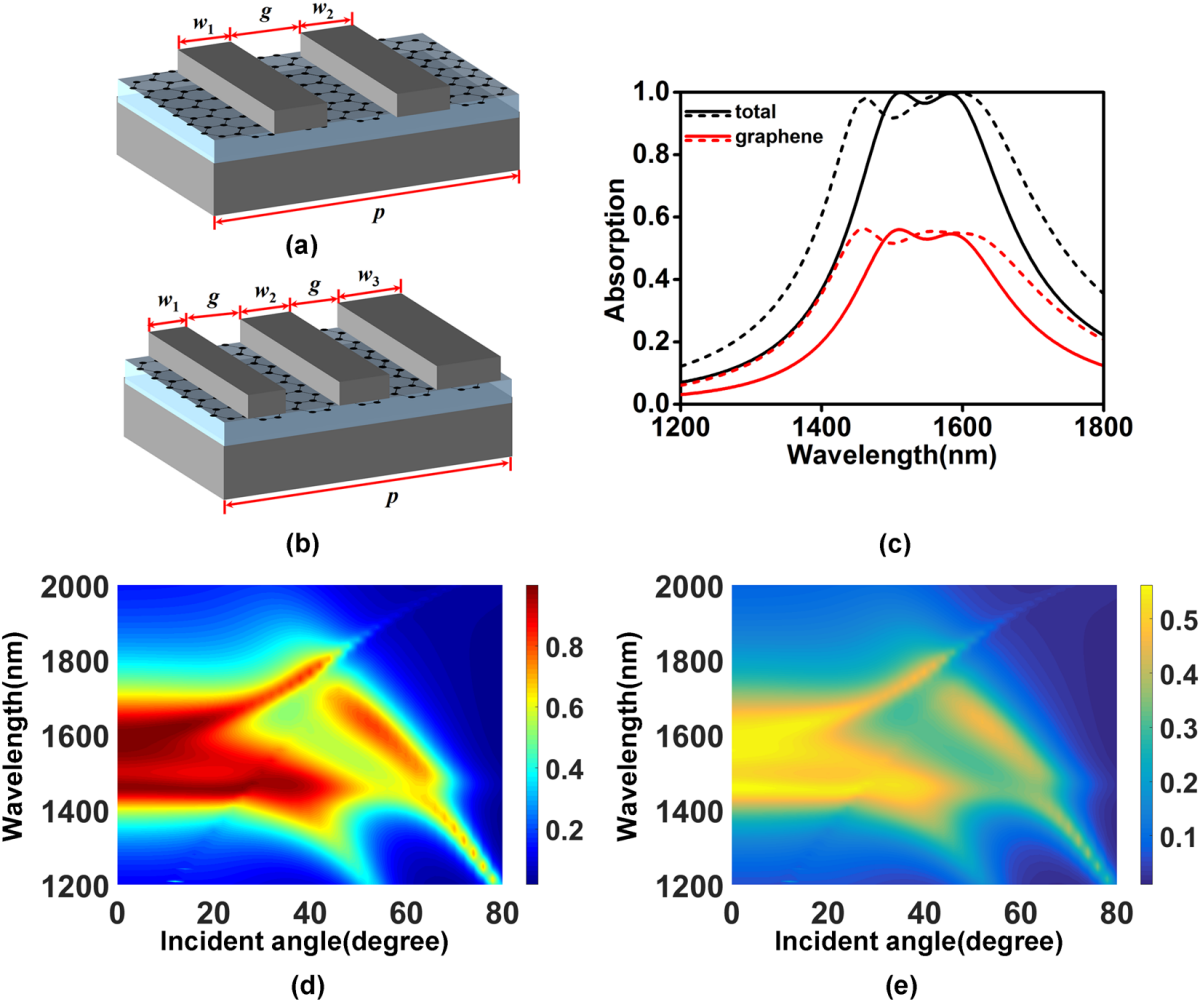
Schematic of the graphene-based absorption structures with (**a**) two and (**b**) three different gratings in one period. (**c**) The absorption spectra of total structure, graphene for the presented structures, where solid lines for (**a**) and dashed lines for (**b**), respectively. Absorption of (**d**) total structure and (**f**) graphene as functions of the wavelength and incident angle for the three-grating structure.

According to the conclusion that the wavelength of magnetic dipole resonance mainly depends on the grating width, we introduce two and three different width gratings in one period to broaden absorption bandwidth. The grating widths and air gap are set as *w*_1_ = 230 nm, *w*_2_ = 250 nm, *g = *100 nm in Fig. 3(a), and *w*_1_ = 220 nm, *w*_2_ = 240 nm, *w*_3_ = 260 nm, *g* = 100 nm in Fig. 3(b). Other geometric parameters are the same as those in Fig. 1(a). Figure 3(c) shows the absorption spectra for the designed two-grating structure (solid lines) and three-grating structure (dashed lines). From Fig. 3(c), we could find that the over-50% absorption bandwidths of total structure and graphene are broadened to 240 nm and 128 nm for two-grating structure, 360 nm and 220 nm for three-grating structure.

The angle-dependent absorption feature of multi-grating resonant structure is similar to that of single-grating resonant structure, as shown in Fig. 3(d–f). The peak absorption and absorption bandwidth are nearly unchanged in the incident angle range from 0 to 20 degree. When the incident angle is over 20 degree, a propagating mode occurs and destroys the stability of the absorption spectra versus incident angle.

**Figure 4:**
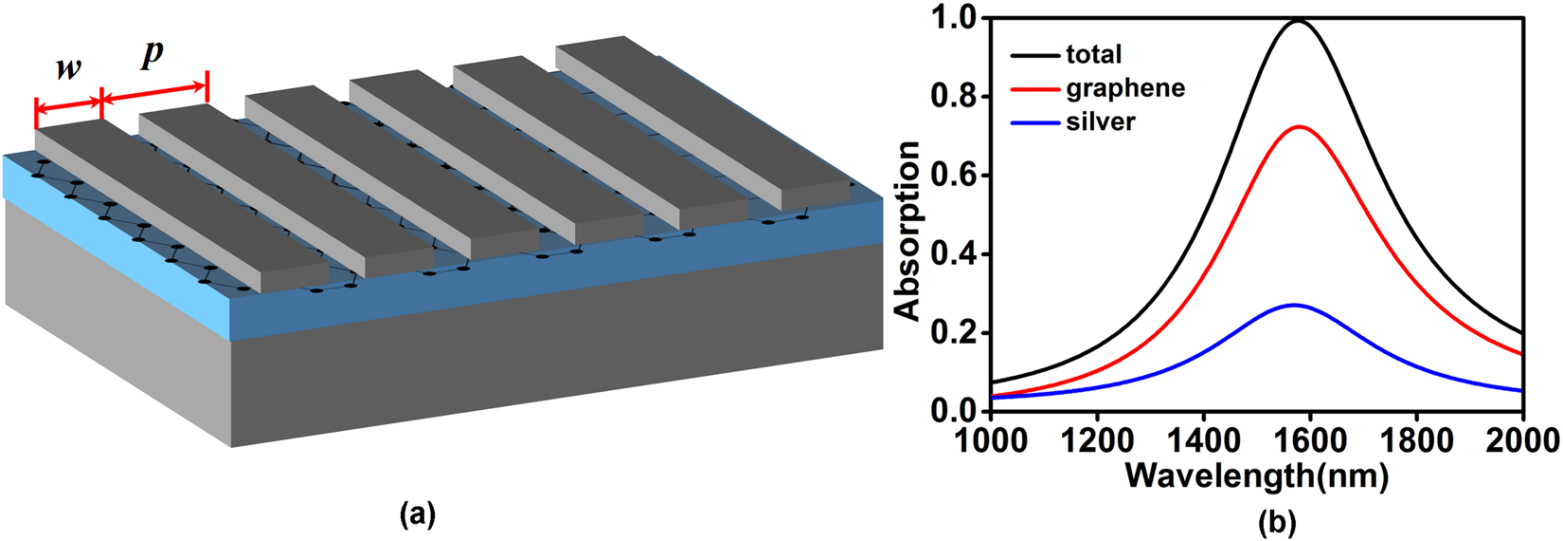
(**a**) Schematic of the graphene-based deep sub-wavelength grating structure. (**b**) The absorption spectra of the total structure, graphene and metal in the (**a**).

Next, we propose a deep sub-wavelength grating structure shown in Fig. 4(a) to enhance the absorption rate and bandwidth of graphene. The geometric parameters of the deep sub-wavelength structure are chosen as follows: *p* = 185 nm, *w* = 165 nm, *h* = 10 nm, *d* = 30 nm, *t* = 100 nm. Figure 4(b) shows the absorption spectra of the designed structure for TM polarization. As shown in Fig. 4(b), when the structure achieves the perfect absorption toward normally incident light with the wavelength of 1540 nm, the peak absorption of graphene approaches 75% with over-50% absorption bandwidth beyond 255 nm, and peak absorption of silver decreases from 45% to 25%, compared to the result in Fig. 1(b).

**Figure 5:**
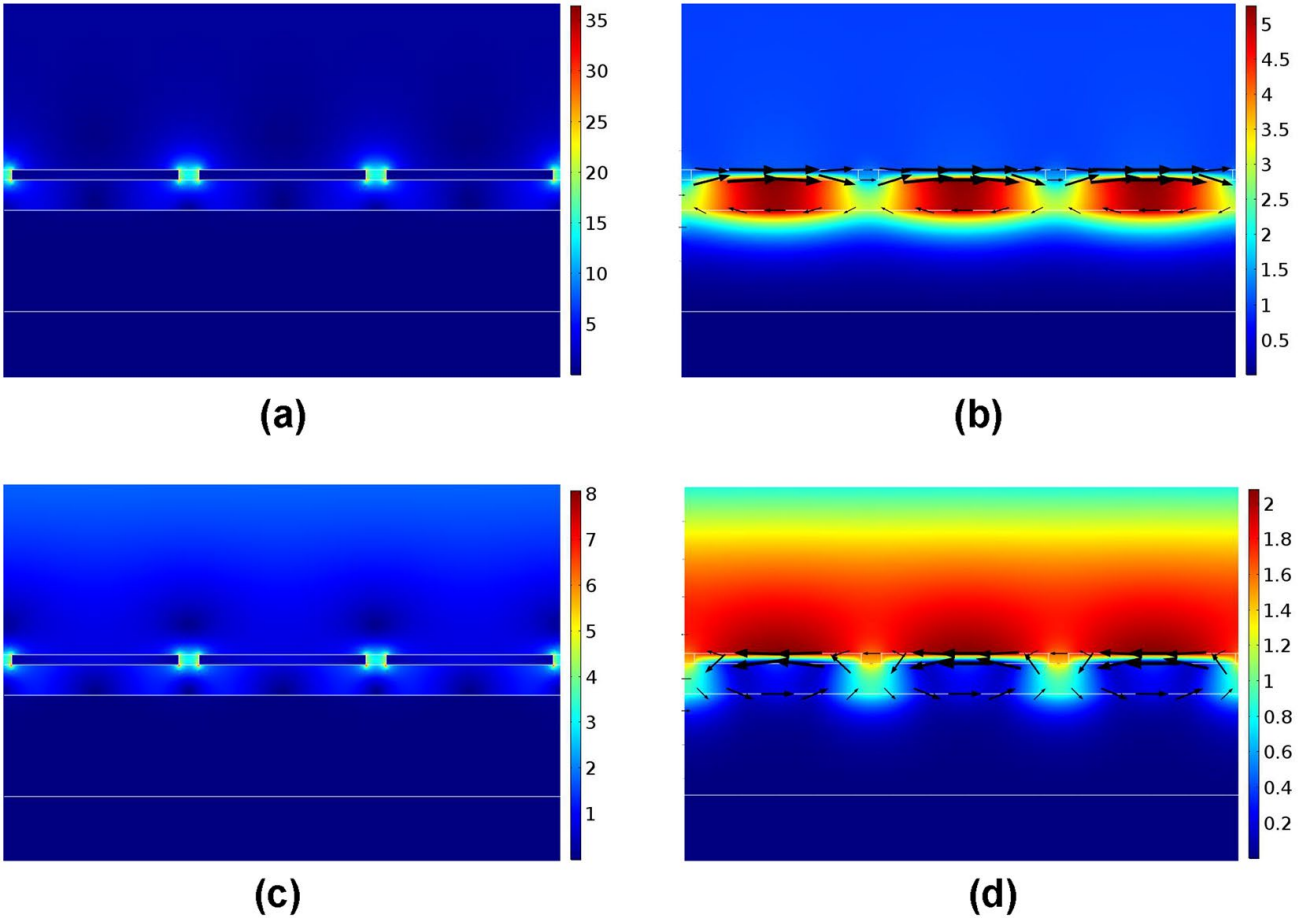
The electric and magnetic field distribution in the deep sub-wavelength structure at on-resonant wavelength (1540 nm) and off-resonant wavelength (1000 nm), where the black arrows represent electric displacement. (**a**) Normalized electric field at 1540 nm. (**b**) Normalized magnetic field at 1540 nm. (**c**) Normalized electric field at 1000 nm. (**d**) Normalized magnetic field at 1000 nm.

In order to reveal the physical origin of the absorption enhancement of graphene, the electromagnetic field distributions at on-resonant and off-resonant wavelengths are plotted in Fig. 5. Figure 5(a,b) show the electric field and magnetic field distributions of the structure at on-resonant wavelength of 1540 nm. As shown in Fig. 5(a), electric field mostly exists in the gaps between silver gratings, which lows the absorption of silver and improves that of graphene. In Fig. 5(b), the direction of electric displacement above and below lossless dielectric layer keeps opposite and almost unchanged along the boundaries of dielectric layer. From Fig. 5(a,b) we can see that the field distributions in the deep sub-wavelength structure at on-resonant wavelength are much different from that in the magnetic dipole resonance structure. This difference originates from the strong coupling of the fields in the adjacent grating units (i.e. magnetic coupling effect), since the gap between gratings in the deep sub-wavelength structure is as small as 20 nm. However, in Fig. 5(c,d), the electromagnetic field is not well confined around the graphene at off-resonant wavelength (1000 nm), resulting in the obvious reduction of graphene absorption.

**Figure 6:**
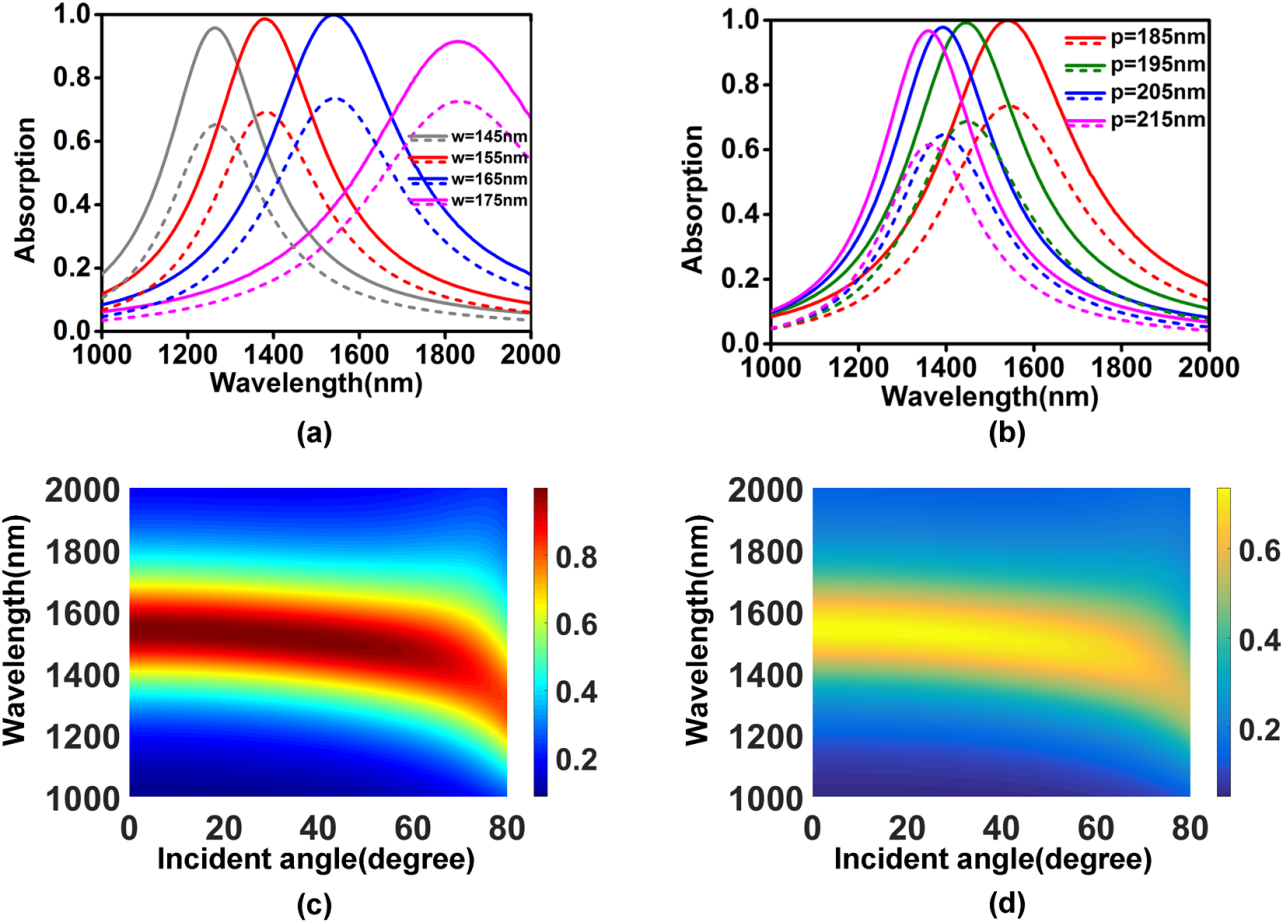
The absorption spectra as functions of (**a**) grating width (**b**) structure period. Other parameters are fixed as in (**a**). Absorption spectra of (**c**) total structure and (**d**) graphene as functions as the wavelength and incident angle for TM polarization.

The influences of geometric parameters on the absorption of total structure and graphene are also calculated and shown in Fig. 6. Although the resonant wavelength of the structure is still dominated by the grating width, the period of the structure also becomes an important factor which affects the resonant wavelength. It can explained from Fig. 5(a,b) where the majority of electromagnetic field concentrates in the gap between gratings, thus the period plays a part in a similar way as the grating width does. In general, the rise of ratio of the grating width to the period causes a red shift on the absorption peak.

The absorption of the total structure and graphene as functions of wavelength and incident angle are shown in Fig. 6(c,d). When the angle varies from 0 to 40 degree, the absorption rate and absorption bandwidth of the structure are very stable, and the absorption peak wavelength only blue-shifts about 25 nm. The peak absorption wavelength and peak absorption rate reduce rapidly with the incident angle over 60 degree.

**Figure 7:**
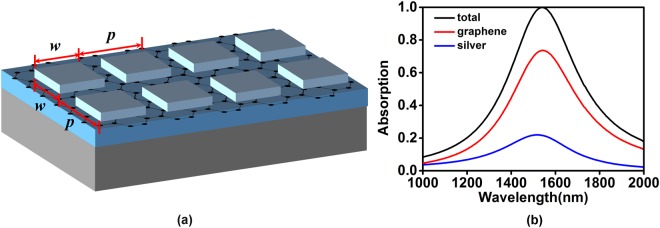
(**a**) Schematic of the graphene-based deep sub-wavelength 2D square plate structure. (**b**) The absorption spectra of the total structure, graphene and metal in the (**a**).

In order to overcome the polarization-dependent disadvantage in above structures, we propose a two-dimension (2D) monolayer-graphene-based structure with periodical square silver plates. The peripheral length of square plate is *w* = 165 nm, 20 nm less than the period of the structure, and other geometric parameters are same as those in Fig. 6(a). The graphene in the proposed structure shown in Fig. 7(a) could absorb 72% energy of the normally incident light with over-50%-absorption bandwidth about 240 nm.

**Figure 8:**
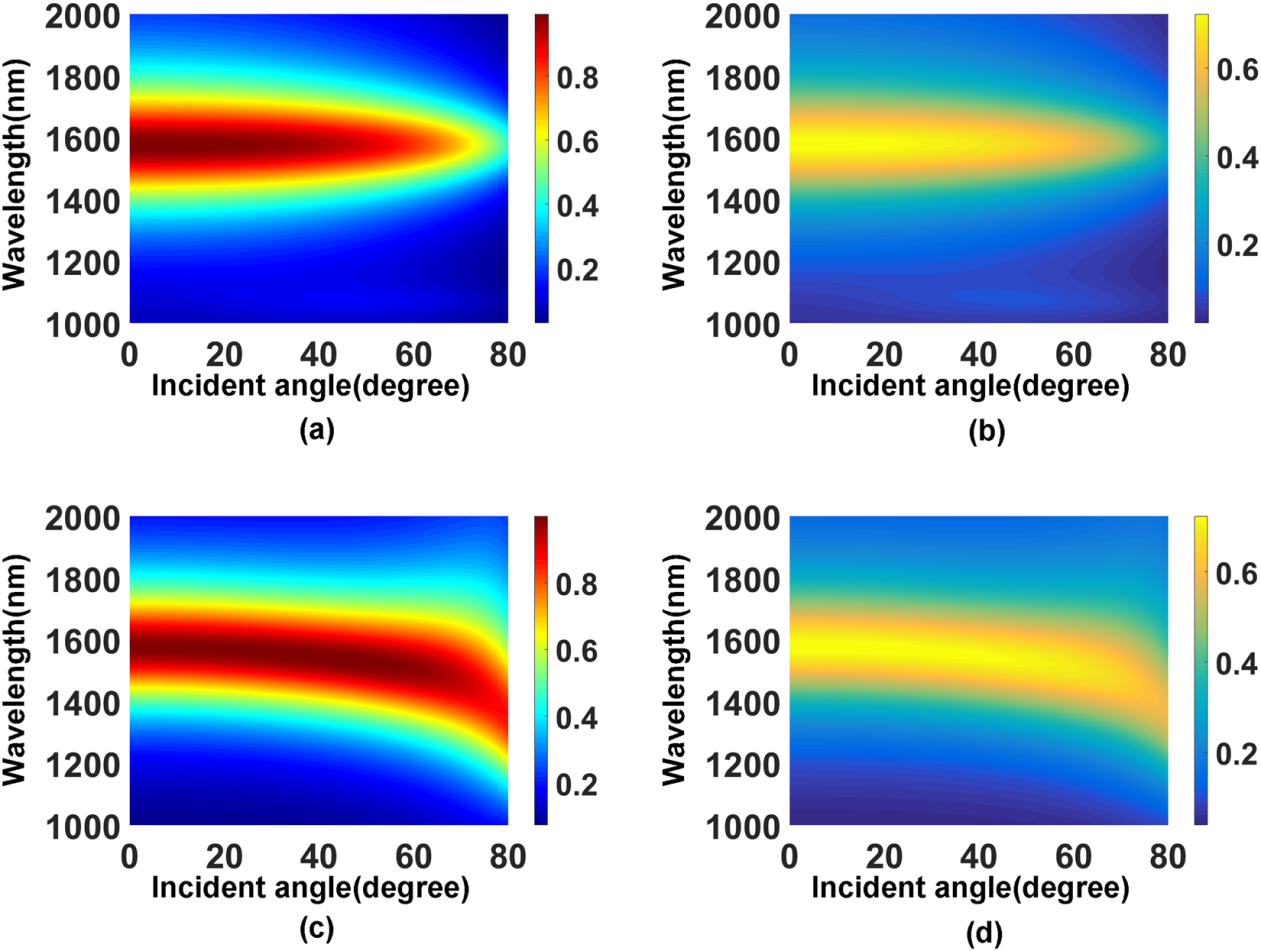
Absorption spectra as functions of the wavelength and incident angle for different polarization. (**a**) Total absorption for TE polarization. (**b**) Total absorption for TM polarization. (**c**) Graphene absorption for TE polarization. (**d**) Graphene absorption for TM polarization.

The influence of geometric parameters on the absorption for the 2D structure is similar to that plotted in the Fig. 6(a,b) for the 1D structure. The most important feature of the 2D structure is the polarization-independent absorption with the incident angle up to nearly 40 degree, as shown in Fig. 8.

## Conclusions

In summary, we proposed and analyzed two kinds of graphene-based perfect absorption structures in the near infrared. The absorption structure with multi-grating in a period by using magnetic dipole resonances could raise the peak absorption rate and over −50% absorption bandwidth of graphene up to 55% and 220 nm. And the absorption structure by utilizing magnetic coupling effects could improve the peak rate and absorption bandwidth to 74% and 255 nm. In addition, the demonstrated structures own ultrathin thickness and high graphene absorption, and can keep stable absorption rate and absorption bandwidth in a large incident angle range, which would be of valuable applications for graphene-based optoelectronic devices.

## Methods

The proposed structures are analyzed using finite-element method-based software (Comsol Multiphysic). In the simulation, the monolayer graphene is regarded as a conductive surface with optical conductance of G_0_ ≈ 6.08 × 10^−5^ Ω^−1^, which corresponds to the absorption of 2.3% for a free standing graphene^1,44,45^. The refractive of alumina is taken as 1.6^46^, and the dielectric constant of silver is given by Drude model as ε(ω) = ε_∞_ − ω_p_^2^/(ω^2^ + iγω) with ε_∞_ = 1.0, ω_p_ = 1.37 × 10^16^ s^−1^, and γ = 6.66 × 10^13^ s^−1^. The damping constant (γ) of silver is doubled compared with the bulk value considering the surface scattering and grain boundary effects of the thin film.
